# Association of Residential Racial and Ethnic Segregation With Legal Intervention Injuries in California

**DOI:** 10.1001/jamanetworkopen.2022.19217

**Published:** 2022-06-29

**Authors:** Cora H. Ormseth, Alyssa C. Mooney, Ojmarrh Mitchell, Renee Y. Hsia

**Affiliations:** 1 School of Medicine, University of California, San Francisco; 2 Department of Epidemiology and Biostatistics, University of California, San Francisco; 3 School of Criminology and Criminal Justice, Arizona State University, Tempe; 4 Philip R. Lee Institute for Health Policy Studies, Department of Emergency Medicine, University of California, San Francisco

## Abstract

**Importance:**

The continued harm of Black individuals in the US by law enforcement officers calls for reform of both law enforcement officers and structural racism embedded in communities.

**Objective:**

To examine the association between county characteristics and racial and ethnic disparities in legal intervention injuries.

**Design, Setting, and Participants:**

This retrospective, cross-sectional study was conducted among 27 671 patients presenting to California hospitals from January 1, 2016, to December 31, 2019, with legal intervention injuries (defined as any injury sustained as a result of an encounter with any law enforcement officer) as identified by *International Statistical Classification of Diseases and Related Health Problems, Tenth Revision* codes.

**Main Outcomes and Measures:**

Legal intervention injuries were classified by *International Statistical Classification of Diseases and Related Health Problems, Tenth Revision* external cause of injury code Y35. Expected injury counts were calculated for each county by multiplying statewide median rates of injury per capita for each age–racial and ethnic group, and then observed to expected injury ratios were measured. The association between county injury ratio, percentage of Black individuals, and residential segregation (measured using an index of dissimilarity) was modeled, stratifying by race and ethnicity.

**Results:**

A total of 27 671 patients (24 159 male patients [87.3%]; 1734 Asian and Pacific Islander [6.3%], 5049 Black [18.2%], 11 250 Hispanic [40.7%], and 9638 White [34.8%]; mean [SD] age, 34.2 [12.5] years) presented with legal intervention injuries in California from 2016 to 2019. Observed to expected injury ratios ranged from 0 to 7 for Black residents and from 0 to 5 for White residents. High observed to expected injury ratios for Black residents (408 observed vs 60 expected; ratio = 7) were clustered around San Francisco Bay Area counties and corresponded with a higher proportion of Black residents. High observed to expected injury ratios for White residents (57 observed vs 11 expected; ratio = 5) clustered around rural northern California counties and corresponded with higher mean percentage of residents with income below the federal poverty level and fewer urban areas.

**Conclusions and Relevance:**

This study suggests that residential segregation may be associated with increased legal intervention injury rates for Black residents of California counties with a large percentage of Black residents. Reform efforts to address racial and ethnic disparities in these injuries should carefully consider and address the legacy of discriminatory policies that has led to segregated communities in California and the United States.

## Key Points

**Question:**

What community characteristics are associated with racial and ethnic disparities in legal intervention injuries in California?

**Findings:**

In this cross-sectional analysis of legal intervention injuries among 27 671 patients in California hospitals, injury counts among Black patients were disproportionately higher compared with their demographic makeup. Increased observed to expected injury ratios for Black patients corresponded with residential segregation and proportion of Black residents, while injuries for White patients corresponded with higher poverty and more rural areas.

**Meaning:**

This study suggests that, to address the disproportionate burden of legal intervention injuries experienced by Black individuals, a history of racist policies that have led to residential segregation must be confronted, and other community characteristics associated with increased risk of injuries must be examined.

## Teaser

This cross-sectional study examines the association between California county characteristics and racial and ethnic disparities in legal intervention injuries.

## Introduction

The continued harm of Black US citizens by law enforcement officers has intensified calls to address law enforcement officer brutality and systemic racism.^1^ Data show that Black people are more likely than White people to experience legal intervention injuries,^1,2,3,4,5,6^ defined as any injury sustained as a result of an encounter with any law enforcement officer.

Hospital administrative data have been used to track these injuries because there is no standardized federal reporting system for legal intervention injuries.^7^ These data show that Black people are disproportionately affected,^2,3,4,5,6^ experiencing legal intervention injuries at a rate 4 to 5 times^3,4^ that of White people and dying at a rate 3 to 6 times^5,8^ that of White people. To address these disparities, states have implemented law enforcement reforms, such as California’s use-of-force standards and the Racial and Identity Profiling Act of 2015.^9^ In parallel, the structural determinants that lead to increased risk of encounters with law enforcement officers must be addressed. Recent studies using government and crowdsourced data have geocoded injuries to examine the characteristics of the communities in which they occur. These studies show that injuries frequently occur in areas of economic deprivation^8^ with household incomes below the national mean,^10^ that income inequality increases the risk of fatal injury for men in racial and ethnic minority groups,^11^ and that racial and ethnic segregation^12^ and a state racism index are associated with racial and ethnic disparities in law enforcement officer shootings.^13^ A limitation of these studies is the use of data sources known to underreport legal intervention injuries.^14,15^

This study is the first, to our knowledge, to analyze hospital data on legal intervention injuries within the context of community characteristics. We used hospital administrative data from 2016 to 2019 to provide updated statistics on injuries in California. We then used census data from the Agency for Healthcare Research and Quality (AHRQ) to examine the association between county-level measures and racial and ethnic disparities in injuries.^16^ We hypothesize that (1) Black residents are disproportionately represented among individuals experiencing legal intervention injuries in California, (2) legal intervention injury rates differ by county, and (3) Black and White residents experience legal intervention injuries differentially by the racial and ethnic and socioeconomic makeup of the county.

## Methods

### Data Source

This is a retrospective analysis of emergency department visits and inpatient admissions for legal intervention injuries as documented by the California Department of Health Care Access and Information (HCAI), formerly known as the Office of Statewide Hospital Planning and Development, from January 1, 2016, to December 31, 2019. Data from California HCAI are mandatorily collected from all licensed hospitals except federally operated hospitals. We followed the Strengthening the Reporting of Observational Studies in Epidemiology (STROBE) guidelines. The study was considered exempt from institutional board review by the University of California, San Francisco, as this is secondary research using data that were recorded in such a manner that the identity of the human participants cannot readily be ascertained directly or through identifiers linked to the participants. Patient consent was waived by the University of California, San Francisco, because no individually identifiable information was available to study personnel.

### Case Ascertainment and Variable Definition

We used *International Statistical Classification of Diseases and Related Health Problems, Tenth Revision* external cause of injury codes (E-codes) to identify injuries resulting from legal intervention.^17^ Each record contains a primary E-code and up to 4 additional E-codes. All records with an E-code of Y35 are classified as legal intervention injuries. Legal intervention injury was defined as “any injury sustained as a result of an encounter with any law enforcement official, serving in any capacity at the time of the encounter, whether on-duty or off-duty. Includes: injury to law enforcement official, suspect and bystander.”^18^ In this analysis, we excluded injuries to law enforcement officers. The following covariates were collected by HCAI: age, sex, self-reported race and ethnicity (American Indian or Alaska Native, Asian, Black or African American, Hispanic, Native Hawaiian or Other Pacific Islander, White, multiracial, and other), hospital disposition, principal language spoken, facility zip code, facility county, principal diagnosis, and other diagnosis.

### County Measures

We linked facility county to the county-level AHRQ social determinants of health data set.^16^ For each county, we included the following AHRQ variables: Gini index of income inequality,^19^ racial segregation measured using the index of dissimilarity, and percentage of residents with income below the federal poverty level. There is a large amount of literature devoted to measures of residential segregation.^20,21^ We chose to use the index of dissimilarity, which has been well validated^22,23^ and measures the extent to which the county population would need to be redistributed across census tracts to achieve a uniform distribution. The index ranges from 0 (complete integration) to 100 (complete segregation) and compares the White vs Black population. Values can be interpreted as the percentage of either group that would have to move to another census tract for the distribution of groups within tracts to equal that of the county.

We used the Surveillance, Epidemiology, and End Results Program age-race-county population estimates to generate the proportion of Asian and Pacific Islander, Black, Hispanic, and White residents in each tract and the population denominators to calculate race- and ethnicity-specific injury rates.^24^ We used the National Center for Health Statistics urban-rural classification scheme for counties, in which 1 represents metropolitan areas with a population of 1 million or more residents and 5 represents micropolitan counties with a population of at least 10 000 but less than 50 000 residents.^25^

### Statistical Analysis

We provide descriptive statistics of patient demographic characteristics, mechanisms of injury, and dispositions of legal intervention injuries in California hospitals from 2016 to 2019. For the analysis of the association between county racial segregation and injuries, we included the 52 counties with a Black population of at least 100 (excludes Alpine, Sierra, Glenn, Mono, Trinity, and Amador counties). Expected injury counts were calculated for Black and White residents of each county by multiplying statewide median rates of injury per capita for each age–racial and ethnic group by the age–racial and ethnic populations in the county. These expected injury counts were compared with actual county injury counts as a ratio of observed to expected injuries. We then reported the socioeconomic characteristics of the counties, by quartile of the ratio of observed to expected injuries by race and ethnicity. Finally, in line with the analysis by Feldman et al,^8^ we constructed negative binomial models of the association between the ratio of observed to expected injuries and the racial and ethnic segregation in the county, controlling for county total population size. This approach models the observed count with an offset term equal to the natural log of the expected injury count, such that results can be interpreted as the ratio of the observed to expected injury counts. We compared the model fit of Poisson and negative binomial models using Akaike information criterion and bayesian information criterion statistics, and we found that the negative binomial specification was a better fit for overdispersion in the data. To test differences in the association of segregation with legal intervention injuries in counties with larger or smaller Black populations, we included an interaction term for the index of dissimilarity and the county percentage of Black residents, each as binary variables for greater or less than the statewide median values of 50.0% and 2.5%, respectively. Statistical analyses were conducted using Stata, version 17 (StataCorp LLC).

## Results

### Injury Characteristics

A total of 27 671 patients (24 159 male patients [87.3%]; 1734 Asian and Pacific Islander [6.3%], 5049 Black [18.2%], 11 250 Hispanic [40.7%], and 9638 White [34.8%]; mean [SD] age, 34.2 [12.5] years) presented with legal intervention injuries in California from 2016 to 2019 (Table 1). Black patients were injured in disproportion to statewide demographic characteristics (18.2% of injuries vs 6.2% of population) compared with Asian and Pacific Islander patients (6.3% of injuries vs 15.6% of population), Hispanic patients (40.7% of injuries vs 39.1% of population), and White patients (34.8% of injuries vs 38.5% of population). These injuries resulted in 26 256 outpatient visits (94.9%), 1336 inpatient admissions (4.8%), and 79 deaths (0.3%). Most patients were male (87.3%), and nearly 1 in 4 patients were younger than 25 years (23.0%). Hispanic, Black, and Asian and Pacific Islander patients were younger than White patients: 28.3% of Hispanic patients, 24.1% of Black patients, and 23.4% of Asian and Pacific Islander patients were younger than 25 years compared with 16.3% of White patients. Nearly half of the population had either a mental health diagnosis (15.4%) or an alcohol or substance use disorder (29.9%). White patients were more likely to have a mental health diagnosis or an alcohol or substance use disorder (54.8%) compared with Asian and Pacific Islander patients (43.7%), Black patients (38.1%), or Hispanic patients (40.6%). Across all groups, the most common principal injury diagnosis was contusion (26.9%), and the leading mechanism of injury was manhandling (29.0%). Firearms were indicated as the principal mechanism in 3.0% of injuries overall and were less likely to be used when White patients were injured (2.5%).

**Table 1.** Characteristics of Patients With Legal Intervention Injuries in California Presenting to the Emergency Department or Hospital, 2016-2019

Characteristic	Patients, No. (%)				
	Asian and Pacific Islander (n = 1734)	Black (n = 5049)	Hispanic (n = 11 250)	White (n = 9638)	Total (N = 27 671)
Age, y					
<18	78 (4.5)	277 (5.5)	728 (6.5)	270 (2.8)	1353 (4.9)
18-24	327 (18.9)	940 (18.6)	2456 (21.8)	1300 (13.5)	5023 (18.2)
25-34	629 (36.3)	1715 (34.0)	4235 (37.6)	3129 (32.5)	9708 (35.1)
35-44	409 (23.6)	1133 (22.4)	2402 (21.4)	2250 (23.3)	6194 (22.4)
45-54	168 (9.7)	614 (12.2)	1013 (9.0)	1616 (16.8)	3411 (12.3)
55-64	75 (4.3)	310 (6.1)	334 (3.0)	809 (8.4)	1528 (5.5)
≥65	48 (2.8)	60 (1.2)	82 (0.7)	264 (2.7)	454 (1.6)
Sex					
Female	228 (13.2)	792 (15.7)	1082 (9.6)	1405 (14.6)	3507 (12.7)
Male	1505 (86.8)	4255 (84.3)	10 168 (90.4)	8231 (85.4)	24 159 (87.3)
Unspecified	1 (0.06)	2 (0.04)	0	2 (0.02)	5 (0.02)
English is primary language	1627 (93.8)	5035 (99.7)	9604 (85.4)	9520 (98.8)	25 786 (93.2)
Comorbidities					
Mental health diagnosis	276 (15.9)	722 (14.3)	1354 (12.0)	1910 (19.8)	4262 (15.4)
Alcohol disorder or SUD	481 (27.7)	1200 (23.8)	3210 (28.5)	3374 (35.0)	8265 (29.9)
Injury diagnosis (top 5)					
Contusion	517 (29.8)	1334 (26.4)	2983 (26.5)	2608 (27.1)	7442 (26.9)
Administrative	189 (10.9)	492 (9.7)	1367 (12.2)	993 (10.3)	3041 (11.0)
Other injuries, external cause	172 (9.9)	556 (11.0)	1293 (11.5)	930 (9.6)	2951 (10.7)
Open wounds of head, neck, trunk	156 (9.0)	454 (9.0)	1136 (10.1)	934 (9.7)	2680 (9.7)
Sprains and strains	113 (6.5)	411 (8.1)	712 (6.3)	645 (6.7)	1881 (6.8)
All other	587 (33.9)	1802 (35.7)	3759 (33.4)	3528 (36.6)	9676 (35.0)
Mechanism of injury (top 5)					
Manhandling	458 (26.4)	1467 (29.1)	3221 (28.6)	2874 (29.8)	8020 (29.0)
Blunt object	75 (4.3)	255 (5.1)	554 (4.9)	428 (4.4)	1312 (4.7)
Sharp object	55 (3.2)	188 (3.7)	313 (2.8)	252 (2.6)	808 (2.9)
Firearm	76 (4.4)	155 (3.1)	363 (3.2)	241 (2.5)	835 (3.0)
Other	835 (48.2)	2415 (47.8)	5390 (47.9)	4448 (46.2)	13 088 (47.3)
Unspecified	235 (13.6)	569 (11.3)	1409 (12.5)	1395 (14.5)	3608 (13.0)
Disposition of patient					
Outpatient	1637 (94.4)	4846 (96.0)	10 683 (95.0)	9090 (94.3)	26 256 (94.9)
Inpatient	90 (5.2)	192 (3.8)	531 (4.7)	523 (5.4)	1336 (4.8)
Died	7 (0.4)	11 (0.2)	36 (0.3)	25 (0.3)	79 (0.3)

Abbreviation: SUD, substance use disorder.

### County Characteristics and Ratios of Observed to Expected Injuries

**Figure 1.**
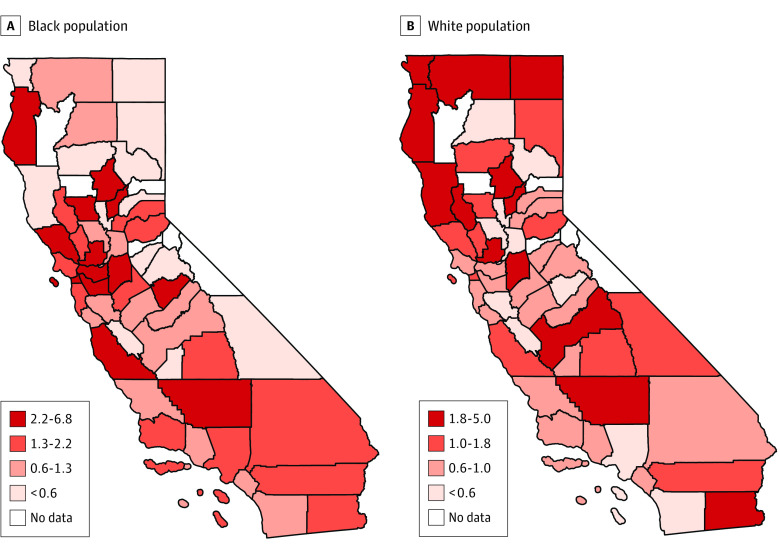
Quartiles of Observed to Expected Injury Ratios for Black and White Residents of California Maps of California showing counties by quartile of observed to expected injury ratios for Black residents (A) and White residents (B). Dark red corresponds with the fourth quartile of injury ratios. High injury ratios for Black residents cluster around San Francisco Bay Area counties, whereas high ratios for White residents cluster in northern counties.

Observed to expected injury ratios ranged from 0 to 7 for Black residents and from 0 to 5 for White residents (eTables 1 and 2 in the Supplement). Figure 1 shows how geographic clustering of observed to expected injury ratios differs by race and ethnicity. San Francisco had the highest ratio of observed to expected injuries among Black residents (408 observed vs 60 expected; ratio = 7), as well as a high level of racial segregation (index of dissimilarity = 58). Del Norte, the most northwestern county in California bordering Oregon, had the highest ratio of observed to expected injuries among White residents (57 observed vs 11 expected; ratio = 5), as well as a lower level of segregation (index of dissimilarity = 47). See eTables 1 and 2 in the Supplement for observed and expected counts, their ratios, and segregation in all counties.

Table 2 and Table 3 compare characteristics of counties across quartiles of the ratio of observed to expected injuries for Black and White residents, respectively. For Black residents, higher injury ratios occurred where a larger mean proportion of the county population was Black. Higher ratios for White residents corresponded with a higher mean percentage of residents with income below the federal poverty level and more rural areas. The inverse was true for Black residents, where higher injury ratios occurred in more urban areas.

**Table 2.** Characteristics of 52 Counties Across Quartiles of the Observed to Expected Injury Ratio Among Black Residents

Characteristic	Mean (SD) value^a^			
	Quartile 1 (range, 0.0-0.53)	Quartile 2 (range, 0.71-1.16)	Quartile 3 (range, 1.36-2.00)	Quartile 4 (range, 2.36-6.83)
Index of dissimilarity, Black vs White patients	56.2 (13.1)	48.4 (6.2)	50.5 (9.1)	49.9 (8.9)
% in poverty	15.7 (4.3)	16.7 (5.2)	15.3 (6.6)	15.3 (4.3)
Gini index of income inequality	0.4 (0.03)	0.5 (0.01)	0.5 (0.02)	0.5 (0.02)
% Black	2.7 (2.4)	3.5 (2.6)	3.7 (2.6)	5.6 (4.3)
% White	66.4 (18.0)	49.8 (17.8)	47.4 (21.7)	50.0 (18.5)
% Hispanic	24.3 (16.2)	34.0 (15.9)	39.6 (21.7)	30.7 (17.3)
NCHS urban-rural classification	4.9 (1.1)	2.7 (1.5)	2.6 (1.2)	3.4 (1.6)

Abbreviation: NCHS, National Center for Health Statistics.

^a^ Quartile ranges represent the minimum and maximum values within each quartile.

**Table 3.** Characteristics of 52 Counties Across Quartiles of the Observed to Expected Injury Ratio Among White Residents

Characteristic	Mean (SD) value^a^			
	Quartile 1 (range, 0.0-0.62)	Quartile 2 (range, 0.66-0.97)	Quartile 3 (range, 0.98-1.70)	Quartile 4 (range, 1.82-5.05)
Index of dissimilarity, Black vs White patients	53.6 (10.9)	47.7 (10.7)	52.1 (9.7)	51.6 (7.7)
% in poverty	13.8 (3.0)	15.1 (5.1)	13.8 (5.2)	20.3 (3.6)
Gini index of income inequality	0.5 (0.03)	0.5 (0.02)	0.5 (0.02)	0.5 (0.02)
% Black	3.5 (3.1)	3.8 (2.8)	3.7 (3.3)	4.4 (3.8)
% White	56.4 (21.7)	51.8 (20.0)	50.2 (17.1)	55.2 (22.6)
% Hispanic	26.2 (15.3)	35.9 (18.3)	34.9 (18.1)	31.5 (21.3)
NCHS urban-rural classification	2.9 (1.9)	3.1 (1.4)	3.3 (1.8)	4.3 (1.1)

Abbreviation: NCHS, National Center for Health Statistics.

^a^ Quartile ranges represent the minimum and maximum values within each quartile.

### Racial Segregation and Ratios of Observed to Expected Injuries

**Figure 2.**
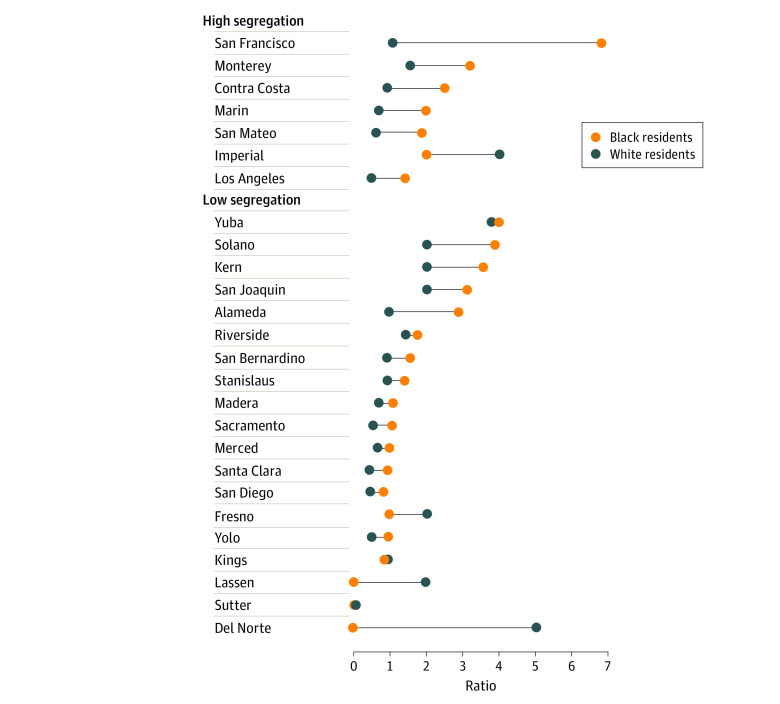
Ratio of Observed to Expected Injuries in Counties With a Black Population Greater Than the State Median (2.5%)

Figure 2 shows observed to expected injury ratios across levels of segregation in counties with a Black population greater than the state median. Observed to expected injury ratios for Black residents were higher than those for White residents in almost every county, and highly segregated counties tended to have higher injury ratios for Black residents and lower injury ratios for White residents.

We then modeled the association between ratios of observed to expected injuries with the interaction of county racial and ethnic segregation and Black population and generated estimates from the model, controlling for county population size. The 52-county sample is underpowered, and we did not detect statistically significant estimates, although the following observations can be noted.

Injury ratios were similar across levels of segregation in counties with a low percentage of Black residents, for both Black and White residents. However, in counties with higher percentages of Black residents, higher levels of segregation may have been associated with higher injury ratios, specifically for Black residents. Among Black residents in counties with a high percentage of Black residents, the injury ratio in counties with higher levels of segregation was 3.05 (95% CI, 1.57-4.52) compared with 1.65 (95% CI, 1.17-2.12) in counties with lower levels of segregation. This finding represents a difference of 1.40 (95% CI, −0.12 to 2.91) across levels of segregation, which is not statistically significant, although it is a substantial effect size that is largely due to data from San Francisco. Conversely, for White residents in counties with a high percentage of Black residents, estimated injury ratios were 1.47 (95% CI, 0.67-2.26) in counties with higher levels of segregation and 1.46 (95% CI, 1.02-1.90) in counties with lower levels of segregation, representing a difference of 0.01 (95% CI, −0.89 to 0.91).

## Discussion

Our study of 27 671 patients with hospital visits for legal intervention injuries in California from 2016 to 2019 found that Black patients had a disproportionately higher rate of injuries compared with their demographic makeup. We geocoded injuries and found that the observed to expected injury ratios for Black and White residents differed by county, with the highest ratios clustering in the San Francisco Bay Area for Black residents and rural northern counties for White residents. In examining county characteristics, we found that the interaction between residential segregation and the Black population of a county corresponded with injury ratios for Black residents. In contrast, rural areas and greater percentages of individuals with income below the federal poverty line corresponded with injury ratios for White residents.

First, we found that Black patients were injured in disproportion to statewide demographic characteristics (18.2% of injuries vs 6.2% of population) compared with Asian and Pacific Islander patients (6.3% of injuries vs 15.6% of population), Hispanic patients (40.7% of injuries vs 39.1% of population), and White patients (34.8% of injuries vs 38.5% of population). This disparity has been well described at the state and national levels. California hospital data from 2005 to 2015 showed higher injury rates for Black patients compared with White patients.^6^ Analysis of the National Trauma Data Bank^3^ and the National Electronic Injury Surveillance System^4^ found that Black patients were injured at a rate 4 to 5 times higher than White patients. Analysis of the National Inpatient Sample and government data found that Black patients made up 27% of individuals with legal intervention injuries, in disproportion to population demographic characteristics and arrests for violent crimes.^2^

Next, we found that ratios of observed to expected injuries for Black and White residents differed by county. Injury ratios were highest for Black residents around San Francisco Bay Area counties and highest for White residents in rural northern California counties. These patterns are consistent with a 2019 analysis of California arrest rates that found total arrest rates were highest in rural counties with poorer economic conditions, but racial disparities were highest in affluent counties with higher educational attainment (in San Francisco, the arrest rate for Black residents was 8 times that of White residents).^26^ The disproportionate arrest rate for Black residents in these counties confers disproportionate risk of injury by law enforcement officers. The San Francisco Bay Area was also implicated in a 2020 nationwide analysis of legal intervention deaths, which found that the San Francisco-Oakland-Hayward metropolitan statistical area had the second-highest rate of fatalities for Black residents (after Oklahoma City, Oklahoma) and the second-highest rate of Black-White inequities of legal intervention deaths (after the Chicago-Naperville-Elgin metropolitan area of Illinois).^27^

Finally, we found differential socioeconomic characteristics of counties with high injury rates for Black vs White residents. For Black residents in counties with a high percentage of Black residents, higher levels of racial segregation were associated with higher rates of legal intervention injuries. This result did not reach statistical significance but showed a notable effect size. High injury rates for White residents corresponded with percentage of residents with income below the federal poverty level and rural areas. These findings are consistent with evidence from crowdsourced and government databases that found that residential segregation and percentage of Black residents in a city were correlated with injury ratios for Black residents^11^ and were associated with Black-White disparities in fatal shootings by law enforcement officers.^12,13^ Data from *The Guardian* showed that fatal shootings by law enforcement officers were highest in areas of economic deprivation and that Black residents were at increased risk in areas with the highest concentrations of White residents.^8^ Here, we contribute similar findings using data that are standardized and mandatorily collected from statewide hospitals.

To illustrate the association between segregation and legal intervention injuries, we examined San Francisco, the county with the highest injury ratios for Black residents. Racist public housing policies, zoning laws, housing covenants, and redlining (intentional discriminatory practices enacted in a geographic area based on a race or ethnicity)^28^ restricted Black residents to the eastern neighborhoods of Hunters Point and the Western Addition. As the Western Addition’s Fillmore District became a vibrant center for Black business and culture, racialized demands for urban renewal targeted it for redevelopment, displacing residents to underresourced neighborhoods, such as Hunters Point and the Tenderloin near downtown.^29^ In 1970, the Black population of San Francisco peaked at 13%, then rapidly decreased as rents increased markedly. In 2019, Black residents made up 6% of the overall San Francisco population but 37% of the unhoused San Francisco population,^30^ and 65% of Black households lived in high-poverty, segregated districts.^31^ These districts have disproportionate rates of stops, searches, arrests, use of force, and killings by the San Francisco Police Department.^29^ Moreover, 81% of formerly unhoused Black residents of San Francisco reported encounters with law enforcement officers.^29^

In 2016, the San Francisco Police Department underwent a review by the Department of Justice after a series of officer-involved shootings and reports of discriminatory text messages exchanged among officers.^32^ The investigation produced 272 recommendations for reform centered on the use of force, bias, community policing practices, accountability, and recruitment practices. However, reform of policing practices in isolation is unlikely to create significant change. Inequities in legal intervention injuries involve not only law enforcement but also inequities in poverty, homelessness, mental illness, and self-medication, many of which can be traced to the legacy of segregation, as we describe. A total of 45.3% of patients injured by law enforcement officers had either a mental health diagnosis or a substance use disorder. Failure of community mental health systems often results in law enforcement officers becoming first responders to individuals experiencing mental health crises,^33^ and inequities within the mental health care system make it such that Black people are more likely to access services through law enforcement involvement.^34^

Our findings reinforce how residential segregation functions as a structural determinant of health^35,36^ by creating concentrated areas of economic deprivation with exposure to law enforcement officers. We linked hospital data on legal intervention injuries to the characteristics of the counties in which they occurred. By mandating the reporting of all emergency department visits and inpatient admissions in California, our data fill a gap in tracking legal intervention injuries, which are currently underreported,^14,15,37,38,39,40,41^ especially for Black residents.^42^ We hope this work will inform policies that respond to the enduring pattern of segregation that perpetuates stark racial disparities in legal intervention injuries.

### Limitations

This study has important limitations. First, we captured only legal intervention injuries that resulted in an emergency department or hospital visit and were correctly coded. Our data, therefore, do not capture deaths pronounced in the field and likely omit less severe injuries. There may be bias in who seeks medical attention (which, if the patient is under custody, is at the discretion of the law enforcement officer and local protocols), who reveals law enforcement involvement in their injury, and how consistently legal intervention injuries are coded and reported.^7^ We do not know if injuries occurred within a jail or prison; these injuries should be excluded to analyze an association with residential segregation.

Second, hospital administrative data do not provide a context for the encounter with a law enforcement officer, such as the reason for the encounter, the race and ethnicity and level of training of the officer, or whether the injured individual was armed. As a result, we are unable to perform more granular analyses for the context in which injuries occurred. Such analysis has been conducted in studies using Racial and Identity Profiling Act data that found that racial and ethnic disparities in use-of-force incidents decrease but persist after controlling for civilian demographic characteristics, the reason for stop, the threat faced by the officer from a weapon, agency, and the closest city.^9^ Analysis of death records from the National Violent Death Reporting System found that Black individuals fatally shot by law enforcement officers were disproportionately unlikely to present an objective threat of deadly force^43^ or have a history of mental illness or substance use, use a weapon, or have a positive toxicology test result compared with White decedents.^44^

Third, our data were collected from California and may not be generalizable to the national level. We chose to use California data to capture geographic identifiers that are unavailable in other national sources (eg, the Nationwide Emergency Department Sample and the AHRQ Healthcare Cost and Utilization Project State Emergency Department Databases and State Inpatient Databases).

## Conclusions

In this cross-sectional study, statewide data from California hospitals suggest that Black residents are disproportionately injured by law enforcement officers. Injury rates for Black and White residents differ by county, and highly segregated counties have higher rates of injury for Black residents. Reducing racial disparities in injuries will require responding to an enduring pattern of residential segregation.

## Notes

Supplement. **eTable 1.** Observed and Expected Legal Intervention Injuries Among Black Residents **eTable 2.** Observed and Expected Legal Intervention Injuries Among White Residents
