# Supplementary material for: Association of Residential Racial and Ethnic Segregation With Legal Intervention Injuries in California
Source: JAMA Netw Open. 2022 Jun 29;5(6):e2219217. doi: 10.1001/jamanetworkopen.2022.19217 (PMC9244606; doi:10.1001/jamanetworkopen.2022.19217)
Supplement: Supplement. — eTable 1. Observed and Expected Legal Intervention Injuries Among Black Residents eTable 2. Observed and Expected Legal Intervention Injuries Among White Residents [file jamanetwopen-e2219217-s001.pdf]

Supplementary Online Content

Ormseth CH, Mooney AC, Mitchell O, Hsia RY. Association of residential racial and ethnic segregation with legal intervention injuries in California. *JAMA Netw Open.* 2022;5(6):e2219217. doi:10.1001/jamanetworkopen.2022.19217

eTable 1. Observed and Expected Legal Intervention Injuries Among Black Residents
eTable 2. Observed and Expected Legal Intervention Injuries Among White Residents

This supplementary material has been provided by the authors to give readers additional information about their work.

© 2022 Ormseth CH et al. *JAMA Network Open.*

eTable 1. Observed and Expected Legal Intervention Injuries Among Black Residents

County	Injury count	Expected count	Ratio of observed-to-expected	Difference in observed and expected	Index of dissimilarity, non-White vs. White	Index of dissimilarity, Black vs. White
San Francisco	408	60	7	348	36	58
Humboldt	17	4	4	13	28	62
Yuba	17	4	4	13	23	46
Solano	295	76	4	219	29	37
Kern	223	62	4	161	27	48
Colusa	1	0	4	1	11	32
Butte	21	7	3	14	27	61
Monterey	50	16	3	34	28	55
San Joaquin	200	64	3	136	29	43
Alameda	621	215	3	406	35	50
Contra Costa	308	122	3	186	36	53
Mariposa	1	0	2	1	23	56
Sonoma	30	13	2	17	30	49
Marin	19	9	2	10	38	52
Lake	3	2	2	1	25	56
San Mateo	44	23	2	21	37	56
Imperial	15	8	2	7	26	52
Placer	17	9	2	8	27	44
Riverside	312	183	2	129	30	40
El Dorado	4	2	2	2	31	62
Tulare	12	8	2	4	15	52
Napa	6	4	2	2	34	66
San Bernardino	340	216	2	124	30	36
Los Angeles	1392	977	1	415	35	58
Stanislaus	27	19	1	8	22	39
Santa Barbara	18	13	1	5	23	45
Santa Cruz	6	5	1	1	37	50
Madera	8	7	1	1	22	42
Siskiyou	1	1	1	0	22	58
Ventura	22	21	1	1	20	47
Sacramento	207	199	1	8	37	43
Orange	77	75	1	2	33	46
Merced	10	10	1	0	23	44
Santa Clara	62	65	1	-3	36	43
San Luis Obispo	8	9	1	-1	24	48
Shasta	3	3	1	0	28	64
San Diego	186	231	1	-45	31	47
Fresno	42	57	1	-15	26	49

© 2022 Ormseth CH et al. *JAMA Network Open.*

Yolo	6	8	1	-2	18	47
Kings	8	15	1	-7	26	33
Lassen	1	5	0	-4	27	45
Sutter	0	3	0	-3	23	37
Del Norte	0	2	0	-2	25	47
Modoc	0	0	0	0	34	51
San Benito	0	1	0	-1	12	53
Tuolumne	0	2	0	-2	18	69
Calaveras	0	1	0	-1	22	66
Tehama	0	1	0	-1	17	61
Mendocino	0	1	0	-1	19	62
Nevada	0	1	0	-1	25	74
Inyo	0	0	0	0	37	62
Plumas	0	0	0	0	20	71

© 2022 Ormseth CH et al. *JAMA Network Open.*

eTable 2. Observed and Expected Legal Intervention Injuries Among White Residents

County	Injury count	Expected count	Ratio of observed-to-expected	Difference in observed and expected	Index of dissimilarity, non-White vs. White	Index of dissimilarity, Black vs. White
Del Norte	57	11	5	46	25	47
Yuba	123	32	4	91	23	46
Imperial	45	12	4	33	26	52
Siskiyou	49	20	2	29	22	58
Modoc	9	4	2	5	34	51
Fresno	463	201	2	262	26	49
Mendocino	77	35	2	42	19	62
Humboldt	157	76	2	81	28	62
Kern	440	215	2	225	27	48
San Joaquin	335	165	2	170	29	43
Solano	246	122	2	124	29	37
Butte	230	118	2	112	27	61
Lake	50	27	2	23	25	56
Lassen	25	15	2	10	27	45
Napa	78	47	2	31	34	66
Monterey	137	87	2	50	28	55
Sonoma	322	211	2	111	30	49
Riverside	842	560	2	282	30	40
Colusa	6	5	1	1	11	32
El Dorado	116	93	1	23	31	62
Tehama	34	28	1	6	17	61
Tulare	104	89	1	15	15	52
Inyo	7	6	1	1	37	62
San Francisco	383	355	1	28	36	58
Alameda	409	414	1	-5	35	50
Ventura	254	259	1	-5	20	47
Placer	179	185	1	-6	27	44
Contra Costa	318	338	1	-20	36	53
San Bernardino	407	438	1	-31	30	36
Stanislaus	148	160	1	-12	22	39
Santa Barbara	126	145	1	-19	23	45
Santa Cruz	99	114	1	-15	37	50
Kings	33	38	1	-5	26	33
Tuolumne	22	27	1	-5	18	69
Calaveras	17	21	1	-4	22	66
Marin	79	109	1	-30	38	52
Madera	24	34	1	-10	22	42
Merced	35	52	1	-17	23	44

© 2022 Ormseth CH et al. *JAMA Network Open.*

Orange	601	917	1	-316	33	46
San Mateo	132	211	1	-79	37	56
Nevada	31	50	1	-19	25	74
San Luis Obispo	82	136	1	-54	24	48
Sacramento	294	509	1	-215	37	43
Shasta	50	95	1	-45	28	64
Los Angeles	1079	2062	1	-983	35	58
Mariposa	4	8	0	-4	23	56
San Diego	573	1171	0	-598	31	47
Yolo	38	82	0	-44	18	47
Santa Clara	201	450	0	-249	36	43
San Benito	1	14	0	-13	12	53
Sutter	0	30	0	-30	23	37
Plumas	0	9	0	-9	20	71

© 2022 Ormseth CH et al. *JAMA Network Open.*
